# A Case Report of a Patient with Turner Syndrome, Multiple Comorbidities, and Pustular Psoriasis: Correlation or Coincidence?

**DOI:** 10.1155/2020/5750309

**Published:** 2020-01-08

**Authors:** Andjela Egger, Andrea Maderal, Hadar Lev-Tov, Olivera Stojadinovic

**Affiliations:** Dr Phillip Frost Department of Dermatology and Cutaneous Surgery, University of Miami Miller School of Medicine, 1600 NW 10th Ave #1140, Miami, FL 33136, USA

## Abstract

Turner syndrome (TS) is one of the most common chromosomal abnormalities. Patients with TS are at an increased risk for the development of metabolic syndrome, hypertension (HTN), diabetes mellitus type II (DM2), hyperlipidemia (HLD), obesity, and cardiovascular disease. The association between psoriasis and the aforementioned conditions including metabolic syndrome, HTN, HLD, obesity, and cardiovascular disease has also been established. Although the mechanism for heightened risk in TS patients is yet to be elucidated, patients suffering from TS and cardiometabolic diseases are likely to be at an even higher risk for developing psoriasis than patients suffering from TS alone. We present a case of a 53-year-old Hispanic woman with a mosaic TS and multiple comorbidities who presented with pustular psoriasis. For this patient, management can be challenging considering her numerous medical comorbidities and the presence of both TS and psoriasis.

## 1. Introduction

Psoriasis is a chronic inflammatory disease commonly seen in dermatologic practice, and its pathogenesis is attributed to Th-1 and Th-17 cell dysregulation among others [1]. Besides the commonly seen rheumatologic issue of psoriatic arthritis, psoriasis has been shown to have an association with metabolic syndrome and its diagnostic components: obesity, insulin resistance, lipid abnormalities, high blood pressure, and related cardiovascular risk factors [2].

Particularly, this association has been found consistently in epidemiologic studies showing that patients with more severe psoriasis have an increased prevalence of metabolic syndrome than patients with mild psoriasis [1]. Turner syndrome (TS) is a genetic condition representing a constellation of characteristic physical features in combination with completely or partially missing X chromosome in a female. TS's associations with autoimmune diseases, including autoimmune skin disorders such as psoriasis [3], lichen planus [4], and alopecia areata [5], have previously been reported in the literature. Further, like psoriasis, TS has also been associated with multiple cardiovascular risks and comorbidities, including metabolic syndrome and DM2 especially in adults [6]. In the current report, we present an adult patient with TS and multiple comorbidities which include metabolic syndrome and DM2 who developed pustular psoriasis. We postulate that patients suffering from TS and cardiometabolic disease may be at a heightened risk for developing psoriasis. Moreover, patients suffering from TS who develop psoriasis may be at an increased risk for developing cardiovascular disease and complications. We believe that clinicians taking care of such patients should be aware of this heightened risk and proactively screen for conditions such as metabolic syndrome and DM2 as early and as efficiently as possible.

## 2. Case Presentation

A 53-year-old Hispanic woman with a mosaic Turner syndrome, presented with a one-week history of a sudden, mildly pruritic widespread rash. Prior to presenting at the University of Miami Department of Dermatology, the patient was seen in the emergency department and was discharged with a triamcinolone ointment which partially alleviated her symptoms. The patient denied a history of skin rashes, upper respiratory infection, constitutional symptoms, or arthralgias. She had numerous medical comorbidities, including hypertension (HTN), coronary artery disease (CAD) status-post stents, history of a cerebral vascular accident, hyperlipidemia (HLD), poorly controlled diabetes mellitus type II, and chronic kidney disease (CKD), which she took several medications for, including atenolol, rosuvastatin, clopidogrel, insulin, aspirin, losartan, ondansetron, and metformin. Yet, she denied any changes to her medication regimen for the past several years. Her past medical history was negative for multiple sclerosis, neurodegenerative disease, hepatitis, tuberculosis, or congestive heart failure.

**Figure 1:**
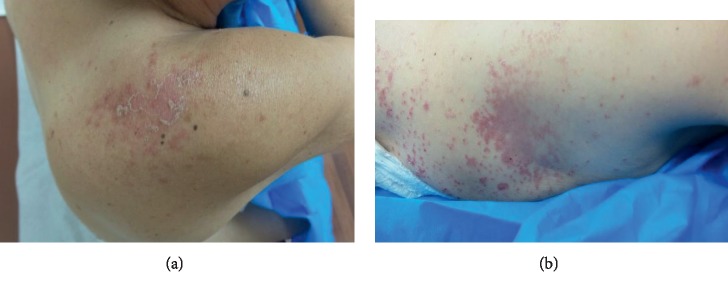
Well-demarcated pink papules and plaques, with fine scale present on the patient's back (a) and chest (b).

The physical examination revealed a generalized eruption of well-demarcated pink papules and plaques, with fine scale and central clearing (Figures 1(a) and 1(b)), involving approximately 10% of the body surface area and mostly lower extremities, back, left axilla, and chest. No lymphadenopathy, nail or mucosal involvement was noted. There was no joint erythema or swelling. Notable laboratory findings included negative antistreptolysin O and anti-DNAse B antibodies and a normal level of serum calcium.

Skin biopsy was obtained and demonstrated scale/crust with a collection of neutrophils between parakeratotic layers, a mild psoriasiform hyperplasia with a reduced granular layer, and mild sparse chronic inflammatory infiltrate in dermis, which is consistent with pustular psoriasis.

The patient appeared to partially respond to topical therapy given at the emergency department, and her more affected areas were treated with clobetasol 0.05% ointment twice a day and her less bothersome areas were treated with triamcinolone 0.1% ointment twice a day. Moreover, treatment also included a narrow band UVB phototherapy which was administered twice a week, while additional work-up including hepatitis panel, quantiferon, CBC, and CMP was obtained in anticipation of potential biologic therapy. Screening labs for possible biologic therapies were negative; however, the patient demonstrated significant improvement and resolution of skin lesions and symptoms after seven narrow-band UVB phototherapy sessions, thus hindering the need for more aggressive treatment.

## 3. Discussion

Turner syndrome is associated with an increased prevalence of autoimmune conditions and increased risk of cardiometabolic diseases [7, 8]. Psoriasis is an immune-mediated disease supported by findings of deregulated response of T-cells, dendritic cells, and proinflammatory cytokines, which improves with immune modulation [9]. TS patients are at an increased risk for developing metabolic syndrome including HTN, DM2, HLD, obesity, and cardiovascular disease [8]. Moreover, TS patients are at an increased risk of developing psoriasis [2, 10, 11]. On the other hand, psoriasis may lead to insulin resistance through various inflammatory cytokines, including the TNF-alpha, which contributes to cell dysfunction and atherogenesis, leading to myocardial infarction and stroke [1]. In cases of TS, however, the mechanism of development of metabolic syndrome and accompanied conditions is yet to be fully understood [11]. Recently, Calcaterra et al. found the prevalence of metabolic syndrome in TS patients to be 4.7% (12.5% in obese and 4.3% in nonobese patients), and metabolic syndrome was associated with visceral adiposity (*P*=0.008). They also reported that the 45,X karyotype is associated with an atherogenic profile [12, 13]. A couple of more recent studies also highlighted the higher prevalence of metabolic comorbidities in the 45,X karyotype [14] as well as the increased risk of metabolic syndrome in TS patients who were older and had weight gain [15]. Particularly, compared to 45,X, mosaicism seems to mitigate the cardiovascular risk factors in TS [16]. TS patients are at a significantly higher risk for developing DM2 than the rest of the population [17]; studies in Denmark have shown that the risk increased 3-4 times [6]. Moreover, there have been some postulations on how this may occur. It appears that the intrinsic genetic defect in TS may play the most important role in the pathogenesis although factors such as autoimmunity and unhealthy lifestyle choices may contribute to a lesser extent. Additionally, metabolic syndrome has been shown to have a significant and positive association with psoriasis development [2]. Considering this, it is possible that patients who suffer from TS and cardiometabolic disease have a higher propensity to develop psoriasis. Moreover, it is also possible that patients who suffer from TS and psoriasis are at an even greater risk of developing or worsening already present cardiometabolic comorbidities, which calls for greater awareness to risk assess and manage cardiovascular disease and metabolic syndrome in this patient population. Ito et al. reported a case of a patient with TS who was suffering from pustular psoriasis and subsequently developed dilated cardiomyopathy hypothesizing that chronic inflammation may be a predisposing factor for dilated cardiomyopathy in this patient population [18]. Davenport proposed management approaches to TS patients [19]; considering our patient suffered from TS and psoriasis, these approaches may have to be enhanced. Similarly, these proposed steps in management should be considered as early as possible as girls with TS are at a greater risk for cardiovascular disease and decreased beta-cell dysfunction than age- and standardized BMI-matched girls [20, 21]. Furthermore, psoriasis management in patients with TS and comorbidities associated with cardiovascular disease may be challenging given the possibility of altered drug metabolism in the liver and via renal clearance [22, 23]. These alterations may exclude therapies such as methotrexate, acitretin, or cyclosporine, especially if the patient has transaminitis, or consequences of CKD.

Our patient, had a history of CKD and a remote history of transaminitis, excluding possible treatment with several aforementioned oral agents. Fortunately, the patient responded well to topical and light therapy. However, a potential treatment with a biological agent was considered initially.

This report describes a case of pustular psoriasis developing in a female with a mosaic TS and multiple comorbidities. This case brings greater awareness to the risks of developing cardiovascular disease and metabolic syndrome in patients suffering from TS and their propensity for psoriasis development. It also highlights the association between TS, psoriasis, and cardiometabolic disease; we propose that patients suffering from TS who develop psoriasis may be at a greater risk for developing or worsening cardiovascular disease and its consequences. Despite having mosaic TS that appears to mitigate the cardiovascular risks compared to the monosomy genotype, our patient suffered from many cardiovascular and metabolic comorbidities and subsequently developed psoriasis. The patient responded well to topical and phototherapy treatment; however, role for biologics warrants further investigation to elucidate the potential benefit versus harm of this treatment modality for this patient population.

If our suspicions, which are grounded in the patient's presentation, are correct, it would substantially adjust future patient care. We emphasize the importance of genotyping patients with TS [24] as well as TS patients with psoriasis receiving closer observation and more aggressive management. As such, this report will bring awareness to this type of presentation, allowing practitioners to further confirm or disconfirm our suspicions.
